# Overexpressing long non-coding RNA OIP5-AS1 ameliorates sepsis-induced lung injury in a rat model via regulating the miR-128-3p/Sirtuin-1 pathway

**DOI:** 10.1080/21655979.2021.1987132

**Published:** 2021-12-01

**Authors:** Haibo Xie, Hanfei Chai, Xiaohong Du, Rongna Cui, Yinan Dong

**Affiliations:** Department of Critical Care Medicine, Zhoushan Maternal and Child Health Hospital, Zhoushan, Zhejiang, China

**Keywords:** Sepsis, lung injury, OIP5-AS1, miR-128-3p, SIRT1

## Abstract

Sepsis, resulting from infections, is a systemic inflammatory response syndrome with a high fatality rate. The present study revolves around probing into the function and molecular mechanism of long non-coding RNA OIP5 antisense RNA 1 (lncRNA OIP5-AS1) in modulating acute lung injury (ALI) mediated by sepsis. Here, a sepsis model was constructed using cecal ligation and puncture (CLP) surgery *in vivo*. The alveolar macrophage cell line NR8383 and the alveolar type II cell line RLE-6TN were dealt with lipopolysaccharide (LPS) for *in-vitro* experiments. We discovered that OIP5-AS1 and Sirtuin1 (SIRT1) were markedly down-regulated in sepsis models elicited by CLP or LPS, while miR-128-3p experienced a dramatic up-regulation. OIP5-AS1 overexpression attenuated NR8383 and RLE-6TN cell apoptosis triggered by LPS and suppressed the expressions of nuclear factor kappa B (NF-κB), inducible nitric oxide synthase (iNOS), interleukin-1β (IL-1β), tumor necrosis factor-α (TNF-α) and interleukin-6 (IL-6) in NR8383 and RLE-6TN cells, whereas miR-128-3p overexpression resulted in the opposite phenomenon. Moreover, OIP5-AS1 overexpression relieved lung edema, lung epithelial cell apoptosis, infiltration of myeloperoxidase (MPO)-labeled polymorphonuclear neutrophils (PMN), inflammatory responses triggered by CLP *in vivo*. Mechanistically, miR-128-3p, which targeted SIRT1, was hobbled by OIP5-AS1. All in all, OIP5-AS1 overexpression enhanced sepsis-induced ALI by modulating the miR-128-3p/SIRT1 pathway, which helps create new insights into sepsis treatment.

## 1 Introduction

Sepsis features life-threatening organ dysfunction arising from dysregulation of the host’s response to infection [1]. In sepsis, the imbalance of pro-inflammatory and anti-inflammatory factors in the body was aroused by a large amount of release and activation from diverse inflammatory mediators comprising tumor necrosis factor-α (TNFα), interleukin-6 (IL-6), interleukin-8 (IL-8), contributing to body immune damage. Of all the organs, the lung is the first and most vulnerable target. Acute lung injury (ALI), a common complication, has a mortality rate up to 25%-30% in 28 days. Notwithstanding, the molecular mechanism of ALI incidence remains a mystery. Clinically, there is no diagnostic marker that may forecast ALI occurrence [2,3]. Therefore, it is imperative to research the molecular mechanism of ALI mediated by sepsis, thus adding to the tools for clinical treatment.

As a type of tissue-specific non-coding RNAs, long non-coding RNAs (lncRNAs), longer than 200 nt, boast inferior stability, and their transcription and dysfunction may influence inflammation development [4,5]. microRNAs (miRNAs), a class of small non-coding RNAs, target a series of messenger RNAs to adjust biological processes, covering cell proliferation, apoptosis, and inflammation [6]. Over the past few years, it has been discovered that lncRNAs adjust relevant proteins to exert a promotional or inhibitory function in sepsis by competitively binding with miRNAs. For instance, in myocardial damage mediated by sepsis, the competitive binding of KCNQ1 opposite strand/antisense transcript 1 (KCNQ1OT1) and miR-192-5p modulates the X-linked inhibitor of apoptosis (XIAP) expression, and KCNQ1OT1 overexpression mitigates myocardial injury stemming from sepsis [7]. lncRNA colorectal neoplasia differentially expressed (CRNDE) exerts a protective impact on liver injury caused by sepsis through miR-126-5p/BCL2 like 2 (BCL2L2) [8]. lncRNA-myocardial infarction associated transcript (MIAT) expression presents an up-regulation in kidney injury incurred by sepsis, whereas miR-29a displays a reverse function. Overexpressed lncRNA-MIAT exacerbates kidney injury resulting from sepsis [9]. Additionally, in lung injury mediated by sepsis, lncRNA taurine up-regulated 1 (TUG1) guards against sepsis-caused ALI through the regulation of the miR-34b-5p/GRB2-associated binding protein 1 (GAB1) axis [10]. These studies confirm that multiple lncRNAs are indispensable to sepsis.

OIP5 antisense RNA 1 (OIP5-AS1), belonging to the lncRNA family members, exerts a pivotal function in regulating tumors like gastric cancer [11] and oral squamous cell carcinoma [12]. miR-128-3p serves as a momentous regulator in umpteen inflammations like Parkinson’s disease [13] and rheumatoid arthritis [14]. The online database ENCORI (http://starbase.sysu.edu.cn) analyzed the underlying binding site between OIP5-AS1 and miR-128-3p, indicating that OIP5-AS1 targeted miR-128-3p. Nevertheless, in sepsis-mediated ALI, whether OIP5-AS1 exerts a corresponding regulatory function by targeting miR-128-3p needs to be further researched.

Sirtuin1 (SIRT1), equipped with deacetylase activity, interacts with a slew of histones and non-histones to transfer the substrate acetyl group to the ADP-ribosyl part of NAD+, thereby maintaining the low acetylation state of histones and the normal gene-level modulation of non-histone in healthy cells [15,16]. miRNAs target SIRT1 and influence the regulation of inflammatory mechanisms. For instance, by targeting SIRT1/eukaryotic translation initiation factor 2A (eIF2a), miR-195 accelerates intestinal epithelial cell apoptosis mediated by LPS [17]. miR-128-3p targets Sirt1 for regulating inflammatory responses of bone marrow mesenchymal stem cell triggered by TNFα [18]. Notwithstanding, whether miR-128-3p modulates sepsis-mediated ALI via targeting SIRT1 remains to be verified.

Here, we uncovered that OIP5-AS1 and SIRT1 were remarkably down-regulated in the rat alveolar macrophage cell line NR8383 and the alveolar type II cell line RLE-6TN that were treated with lipopolysaccharide (LPS), coupled with a stark up-regulation of miR-128-3p. OIP5-AS1 overexpression greatly dampened NR8383 and RLE-6TN cell apoptosis and inflammation mediated by LPS. A sepsis-elicited ALI model was set up through cecal ligation and puncture (CLP), displaying that OIP5-AS1 was vehemently down-regulated in the CLP rat model. Therefore, we conjectured that OIP5-AS1 modulated the miR-128-3p/SIRT1 axis to guard against ALI incurred by sepsis. In summary, the paper is dedicated to a research on the regulatory function and mechanism of OIP5-AS1 in sepsis-caused ALI, in the hope that novel efficacious targets can be developed for ALI mediated by sepsis.

## 2 Materials and methods

### 2.1 Animals and grouping

Twenty-four male Sprague-Dawley (SD) rats, 240–260 g in weight, were supplied by the animal center of Zhejiang University. They were reared for 10 days under controlled environmental conditions and given free access to food and water for adaptation to the surroundings. All experiments, granted by the Animal Experiment Ethics Review Committee of the Pharmaceutical Research Institute of Zhoushan maternal and Child Health hospital, were implemented in line with the laboratory animal care and use guidelines of the National Institutes of Health (No. 85–23, revised 1996). The rats randomly fell into four groups (6 rats each): the Sham group, the CLP group, the CLP+Ad-GFP group, and the CLP+Ad-OIP5-AS1 group. The adenovirus vector, associated with the enhanced green fluorescent protein gene, was bought from Life Technologies (Shanghai, China). Gateway ™ LR Clonase II Enzyme Mix (Invitrogen, Carlsbad, USA) was adopted to transfer OIP5-AS1 complementary DNA (cDNA) or negative control (NC) to Ad-OIP5-AS1 (or Ad-GFP). Afterward, 20 μL of the adenovirus solution (10^7^ particles/μL) was injected into the tail vein one week prior to the CLP/sham operation.

### 2.2 Cecal ligation and puncture model

We carried out a CLP operation to engineer a sepsis-ALI rat model referring to [19] with fewer changes. Briefly, the anesthetization in animals was implemented through the intraperitoneal injection of pentobarbital sodium (30 mg/kg body weight, Sigma Aldrich, St. Louis, USA), with the fixation of animals in the supine position on an operating table. The rats were cut on the abdomen being subjected to a 0.4 cm longitudinal midline incision, with the cecum exposed and ligated with a 3–0 silk thread 1 cm from the end. The cecum was pierced once with a 20-gauge needle 0.5 cm from the ligation point. After gently squeezing a handful of feces out of the cecum, we repositioned the intestine in the abdominal cavity. Promptly subsequent to the operation, fluid resuscitation was done by subcutaneously transfusing normal saline. The sham operation group adopted the parallel operation method barring cecal ligation or puncture. Upon the completion of CLP operation, the rats were intraperitoneally transfused with normal saline, waiting to be observed in terms of survival. Forty-eight hours after modeling, the rats were killed utilizing pentobarbital sodium (100 mg/kg body weight), with their lung tissues immediately harvested. As all lungs were resected, the right lung tissues were taken for the measurement of pulmonary edema via the dry/wet method, while the left lungs were utilized for histological and immunohistochemistry analysis.

### 2.3 Hematoxylin and eosin staining

The left lung tissues were immobilized with 4% paraformaldehyde at 4°C for 24 h. The tissues were made dry with ethanol, embedded in paraffin, sectioned into slices 5 μm in thickness employing a microtome (Leica RM2125RT, Leica, Nussloch, Germany). The paraffin was removed from the sections with the use of xylene, and ethanol with gradient concentrations (95%, 90%, 80%, and 70%) was taken to dehydrate the slices. Put in distilled water, the obtained sections were dyed with the hematoxylin aqueous solution (Beyotime, Shanghai, China) for 5 minutes. After being rinsed in tap water, the sections were subjected to several seconds’ differentiation with 1% alcohol hydrochloride. They were flushed by tap water again and then transformed back to blue with 0.6% ammonia. After being slowly washed in running water for 10 minutes, the slices were dyed with eosin for 3 min or so. At last, they were dehydrated by anhydrous ethanol, made transparent with xylene, and sealed [20]. An Olympus BX 51 optical microscope (Olympus Corporation, Tokyo, Japan) was exploited to observe the sections at 200 X magnification.

### 2.4 Immunohistochemistry (IHC)

IHC was conducted to examine SIRT1 and MPO in the left lung tissues [21]. The lung tissues were collected, immobilized in 4% paraformaldehyde (at 4°C overnight), embedded in paraffin, sliced up (5 μm thick), dewaxed with dimethylbenzene and hydrated employing gradient ethanol. With the endogenous peroxide blocked with 1% hydrogen peroxide (H_2_O_2_) for 5 min, the sections, flushed 3 times with phosphate buffered saline (PBS), were sealed for 1 h with an immunostaining blocking solution for incubation overnight at 4°C along with the anti-SIRT1 antibody (1: 200, Abcam, ab110304, MA, USA) and the anti-MPO antibody (1: 200, Abcam, ab208670, MA, USA). After being flushed in PBS, these were incubated along with antiserum linked by biotin for 1 h at ambient temperature. The sections were rinsed again, dyed with 3,3-diaminobenzidine hydrochloride for 1 min, flushed with double distilled water, stained with hematoxylin for 1 min, and at last examined by a microscope (Olympus, Japan).

### 2.5 The measurement of pulmonary edema via the dry/wet method

The dry/wet weight method was adopted for the determination of the lung tissue moisture content in rats [22]. The right lung tissues of each group, taken out for a quick measurement of the wet weight, were kept in an oven at a high temperature of 70°C and made dry for 48 h. The weight of dried lung tissues was measured. The dry/wet ratio of the lung tissues is presented as dry weight/wet weight×100%.

### 2.6 Cell culture and transfection

The rat alveolar macrophage cell line NR8383 and the alveolar type II cell line RLE-6TN, acquired from the American Type Culture Collection (ATCC, Manassas, USA), were grown in an Ham’s F-12 K medium (Gibco, Catalog.No. 21,127,022) incorporating 10% fetal bovine serum (FBS, Gibco, Catalog.No. 10,100,147) as a mixed population of adherent cells and non-adherent cells. These cells were cultivated in Ham’s F-12 K medium comprising 2 mm L-glutamine, 40 U/ml penicillin, and 40 μg/ml streptomycin. Meanwhile, the medium was added with 5 μg/ml insulin, 25 μg/ml transferrin, 10 μg/ml bovine pituitary extract, 2.5 ng/ml insulin-like growth factor (IGF), and 2.5 ng/ml epidermal growth factor (EGF). All cells were cultivated to confluence in a humidified incubator at 37°C. Twenty-four hour prior to transfection, NR8383 cells in the logarithmic growth phase were seeded into 96-well microplates to achieve a fusion rate at 60–80%. In keeping with the manufacturer’s protocol, FuGENE® reagent (Promega, Madison, WI, USA) was applied for the transient transfection of plasmids (covering OIP5-AS1 overexpression plasmid or OIP5-AS1-NC) or miR-128-3p mimics and miR-NC (GenePharma, Shanghai, China) into NR8383 and RLE-6TN cells for 48 h, respectively [23]. Six hours following stimulation with lipopolysaccharide (LPS, Sigma-Aldrich) of different concentrations (25, 50, 100 ng/mL), NR8383 or RLE-6TN cells were harvested for further experiments.

### 2.7 Quantitative reverse transcription-polymerase chain reaction (qRT-PCR)

TRIzol reagent (Invitrogen, Carlsbad, CA, USA) was taken to extract total RNA out of the rat lung homogenate, NR8383 and RLE-6TN cells. Spectrophotometry was deployed to check the RNA purity and concentration, with the absorbance ratio of 260 nm/280 nm taken to verify whether the RNA purity was qualified. 2 µg of the RNA samples were reverse-transcribed into cDNA with the assistance of the RevertAid First Strand cDNA Synthesis Kit (Thermo Fisher Scientific, Waltham, Ma, USA). Reverse transcription reaction was implemented as instructed by the reverse transcription kit under these conditions: 10 min at 70°C, 5 min on ice, 60 min at 42°C, 5 min at 95°C, and 5 min at 0°C. The SYBR® Premix-Ex-Taq ™ (Takara, TX, USA) and ABI7300 systems were introduced for qRT-PCR. The total volume of the PCR system was set to 30 µL. 300 ng cDNA, 6 µL of 5× SYBR, 0.5 µL of upstream primers, and 0.5 µL of downstream primers were added to each well, followed by the addition of sterilized distilled water to make the total volume reach 30 µL. The amplification procedures encompassed 10 min initial denaturation at 95°C, followed by 45 cycles at 95°C for 10 sec, 60°C for 30 sec, and 85°C for 20 sec. All fluorescence data were transformed into relative quantification. β-actin was taken as the endogenous control of OIP5-AS1, while U6 was adopted as the endogenous control of miR-128-3p. Statistical analysis was performed applying the 2^−ΔΔCt^ method [24]. Each experiment was duplicated 3 times. RNA primer sequences are detailed as follows:

OIP5-AS1 Forward: 5ʹ-TGCGAAGATGGCGGAGTAAG-3ʹ;

OIP5-AS1 Reverse: 5ʹ-TAGTTCCTCTCCTCTGGCCG-3ʹ;

miR-128-3p Forward: 5ʹ-CGGGCTCACAGTGAACCGG-3ʹ;

miR-128-3p Reverse: 5ʹ-CAGCCACAAAAGAGCACAAT-3ʹ;

U6 Forward: 5ʹ-TGCGGGTGCTCGCTTCGGCAGC-3ʹ;

U6 Reverse: 5ʹ-CCAGTGCAGGGTCCGAGGT-3ʹ;

β-actin Forward: 5ʹ-CAGAGCCTCGCCTTTGCC-3ʹ;

β-actin Reverse: 5ʹ-GTCGCCCACATAGGAATC-3ʹ.

### 2.8 Western blot

The rat lung tissue homogenate, NR8383 and RLE-6TN cells were collected. The RIPA protein lysis buffer (Roche, Basel, Switzerland) was affiliated for total protein isolation. 50 µg of the total protein sampled in 12% polyacrylamide gel was electrophoresed at 100 V for 2 h and then electrically moved onto polyvinylidene fluoride (PVDF) membranes. After being sealed with 5% nonfat milk powder for an hour at regular atmospheric temperature, the membranes were flushed 3 times with TBST (10 min each) for the overnight incubation at 4°C along with the anti-SIRT1 antibody (1: 1000, Abcam, ab110304, MA, USA), anti-p-NF-κB antibody (phospho S536) (Abcam ab86299, 1: 1000), anti-NF-κB antibody (1: 1000, Abcam, ab32536, MA, USA), anti-iNOS antibody (1: 1000, Abcam, ab15323, MA, USA), anti-Bax antibody (1: 1000, Abcam, ab32503, MA, USA), anti-Bcl2 antibody (1: 1000, Abcam, ab182858, MA, USA), and anti-Caspase3 antibody (1: 1000, Abcam, ab13847, MA, USA). Following TBST washing, the membranes were incubated along with the anti-rabbit or anti-mouse secondary antibody (1: 3000) labeled by horseradish peroxidase (HRP) for 1 h [25]. TBST was applied to rinse the membranes three times (10 min each). At last, Western blot special reagent (Invitrogen) was employed for color imaging; the gray value of each protein was analyzed by Image J.

### 2.9 Cell counting kit-8 (CCK-8) assay

NR8383 and RLE-6TN cells in the logarithmic growth stage was taken for digestion, centrifugation, and counting. After being inoculated into a 96-well plate with a density of 2 × 10^4^/ml (100 μL per well), the cells were cultivated in a 37°C incubator with 5% CO_2_ for 24 h. Once the above procedures were accomplished, the culture solution was removed. Subsequent to cell treatment following the test group, 10 μL of CCK-8 solution (Beyotime Biotechnology, Shanghai, China) was given to each well for one-hour further incubation. Thermo Scientific™ Varioskan™ LUX (VL0L00D0, Thermo Fisher Scientific, Shanghai, China) was introduced to examine each well’s absorbance value at 450 nm wavelength [26]. Four parallel holes were set in each group. The assay was duplicated three times.

### 2.10 Flow cytometry for apoptosis detection

NR8383 and RLE-6TN cells were collected through centrifugation (1500 r/min, 3 min). The collected cells operated as per the instructions of the apoptosis detection kit (Shanghai Aladdin Biological Reagent Co., Ltd.) were flushed twice with PBS. With the addition of 400 μL pre-chilled PBS, 10 μL of AnnexinV-FITC and 5 μL of PI were administered to the cell samples. Following cell cultivation in the dark at 4°C for 30 min, the Invitrogen Attune NXT flow cytometry (Catalog. number was A24863, Thermo Fisher Scientific, Shanghai, China) was manipulated to monitor apoptosis [27]. Through computer algorithm processing, the percentage of apoptosis was worked out.

### 2.11 Enzyme linked immunosorbent assay (ELISA)

A centrifugation (15,000 r/min, 20 min) was implemented at 4°C for acquiring lung tissue homogenates. After being digested by 0.25% trypsin (Beyotime, Shanghai, China), NR8383 and RLE-6TN cells were centrifuged at 1000 r/min and 4°C for 10 min to harvest the cell supernatant. The lung tissue supernatant or cell supernatant was taken for measurement. All steps were strictly in line with the instructions supplied by ELISA kits to examine the content of IL-1β (Catalog.No. H052-1), TNFα (Catalog.No. H002), and IL-6 (Catalog.No. H007-1-1) [28]. The previously described test kits were purchased from Nanjing Jiancheng Bioengineering Institute, China.

### 2.12 Dual luciferase reporter assay

The DNA sequence, which was amplified, was cloned into pmirGLO dual-luciferase vector (Promega, Madison, WI, USA) for building reporter vectors of wild type (WT) OIP5-AS1, mutant type (MUT) OIP5-AS1, wild type (WT) SIRT1-3ʹUTR and mutant (MUT) SIRT1-3ʹUTR, respectively. We positioned NR8383 cells in 24-well plates overnight and applied Lipofectamine 2000 (Invitrogen, USA) to transfect the above luciferase reporter vectors and miR-128-3p mimics or miR-NC into the cells [29]. Forty-eight hours after transfection, the luciferase activity test was conducted using a dual-luciferase reporter gene assay system (Promega, Madison, WI, USA).

### 2.13 RNA immunoprecipitation (RIP) assay

To ascertain the interrelationship between OIP5-AS1 and miR-128-3p, as well as SIRT1 and miR-128-3p, we adopted Magna-RNA binding protein immunoprecipitation kit (Millipore, Bedford, MA, USA) for RIP detection. Generally, NR8383 cells at 80% confluence were acquired and lysed in complete RIP lysis buffer. NR8383 cell lines (1 × 10^5^ cells) were co-immunized with RIP buffer, which contained anti-argonaute2 (Ago2) antibody (Millipore) or magnetic beads of negative control normal rat IgG (Millipore). The specimen was digested with proteinase K to isolate the immunoprecipitated RNA [30]. Finally, RT-qPCR was used for determining OIP5-AS1 and SIRT1 enrichment in the immunoprecipitated RNA.

### 2.14 Statistical analysis

Statistical analysis of data was implemented utilizing SPSS software (version 20.0, Chicago, USA). All data were expressed as mean ± standard deviation. Shaprio-Wilk tests were used to assess the normality of data distributions. We employed student t test for statistical analysis between two groups. Multi-group comparisons were performed using one-way ANOVA followed by Tukey’s post hoc test. We adopted the Pearson correlation test to analyze the correlation test. It was considered statistically different when the difference was shown as *P* < 0.05.

## 3 Results

### 3.1 Expression characteristics of OIP5-AS1 and miR-128-3p in sepsis models

**Figure 1.**
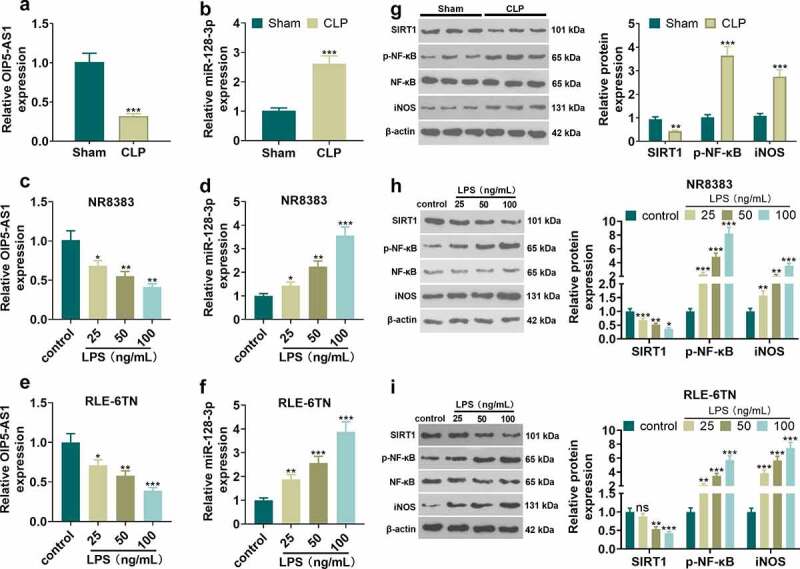
Expression characteristics of OIP5-AS1 and miR-128-3p in the sepsis model. CLP surgery was used to induce septic rat model. 48 hours after the construction of the mode, the lung tissues were isolated and subjected to further experiments. NR8383 and RLE-6TN cells were treated with LPS (25, 50, 100 ng/mL) for 6 hours. A-E. qRT-PCR was taken to determine OIP5-AS1 and miR-128-3p relative expression in septic rats (a-b) and LPS-treated NR8383 and RLE-6TN cells (c-f). G-I. The relative expression of SIRT1, p-NF-κB and iNOS in septic rats (g) or was LPS-treated NR8383 and RLE-6TN cells (h-i) was tested applying Western blot. *P < 0.05, ** *P* < 0.01, *** *P* < 0.001 vs. sham group or control group. N = 3

To probe the role of OIP5-AS1 and miR-128-3p in sepsis, we conducted CLP surgery on 24 SD rats to build a sepsis model *in vivo*, and constructed a sepsis cell model on NR8383 and RLE-6TN cells employing LPS. The relative expression of OIP5-AS1 and miR-128-3p was tested adopting the qRT-PCR method. The results manifested that OIP5-AS1 expression was markedly downregulated in sepsis rat model and cell model (NR8383 and RLE-6TN), while miR-128-3p expression was elevated (P < 0.05 vs. Sham group or control group, Figure 1a-f). We took Western to test the relative expression of SIRT1, p-NF-κB and iNOS. The results illustrated that SIRT1 was lowered in sepsis-mediated rats and LPS-treated cells (NR8383 and RLE-6TN) while p-NF-κB and iNOS were heightened (P < 0.05 vs. Sham group or control group, Figure 1g-i), The above results indicated that OIP5-AS1 and SIRT1 expressions were in down-regulation, and miR-128-3p expression was in upregulation during sepsis development

### 3.2 OIP5-AS1 overexpression attenuates lung epithelial cell damage and macrophage inflammation

**Figure 2.**
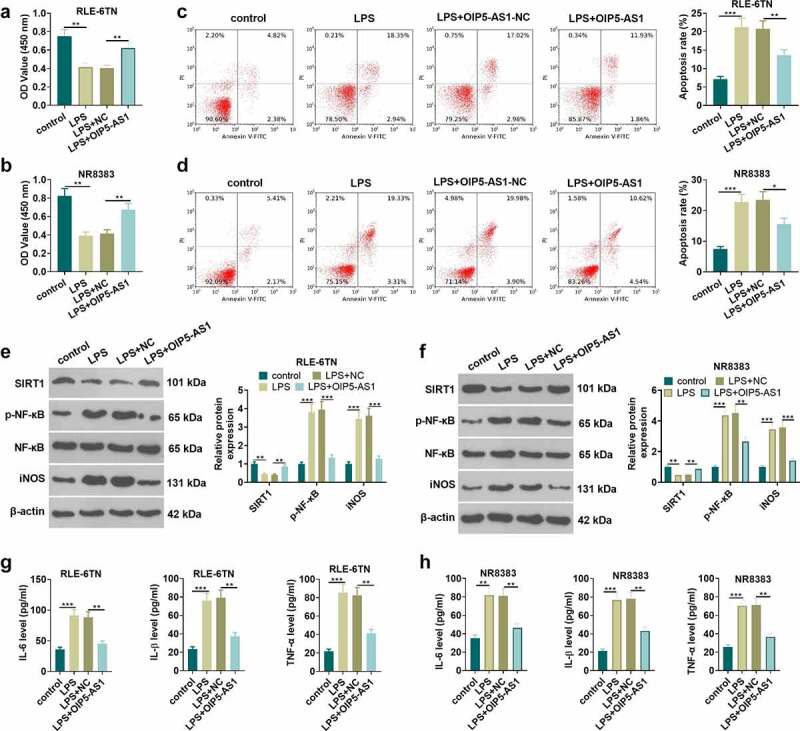
Overexpressing OIP5-AS1 attenuated lung epithelial cell damage and macrophage inflammation. RLE-6TN cells and NR8383 cells were transfected with OIP5-AS1 overexpression plasmids or the negative control (NC), and then both were treated with 100 ng/mL LPS for 6 hours. A-D RLE-6TN cell and NR8383 cell viability and apoptosis were examined adopting CCK8 assay (a-b) and flow cytometry (c-d), respectively. E-F. The test of the relative expression of SIRT1, p-NF-κB and iNOS in RLE-6TN cells and NR8383 cells was carried out using Western blot. (g, h) We employed the ELISA method for detecting the levels of related inflammatory factors IL-6, IL-1β, and TNFα in RLE-6TN cell and NR8383 cell. * *P* < 0.05, ** *P* < 0.01, *** *P* < 0.001. N = 3

For investigating the protective effect of OIP5-AS1 against sepsis, the OIP5-AS1 overexpression plasmids or the negative vector (NC) were transfected into RLE-6TN and NR8383 cells. The viability and apoptosis of RLE-6TN and NR8383 cells were examined using the CCK8 method and flow cytometry, respectively. The results manifested that LPS induced cell viability inhibition and the increase of apoptosis rate. Following OIP5-AS1 overexpression plasmids transfection, enhanced cell viability and restrained cell apoptosis were observed (*P* < 0.05 vs.LPS+NC group, Figure 2a-d). Western blot was utilized to measure the relative expression of SIRT1, p-NF-κB and iNOS in RLE-6TN and NR8383 cells. We found that OIP5-AS1 overexpression weakened the reduction of SIRT1 and the upregulation of p-NF-κB and iNOS following LPS treatment (*P* < 0.05 vs.LPS+NC group, Figure 2e, f). The levels of inflammatory associated factors, including IL-1β, TNFα and IL-6 in LE-6TN and NR8383 cells were examined by ELISA. The data uncovered that LPS accelerated the release of IL-6, IL-1β, and TNF-α from RLE-6TN and NR8383 cells, while OIP5-AS1 overexpression led to the reduction of the expression of related inflammatory factors (*P* < 0.05, Figure 2g, h). The data revealed that overexpressing OIP5-AS1 lessens the damage of lung epithelial cell and macrophages and suppresses the release of inflammatory factors.

### 3.3 *Overexpressing OIP5-AS1 attenuates lung injury* in vivo

**Figure 3.**
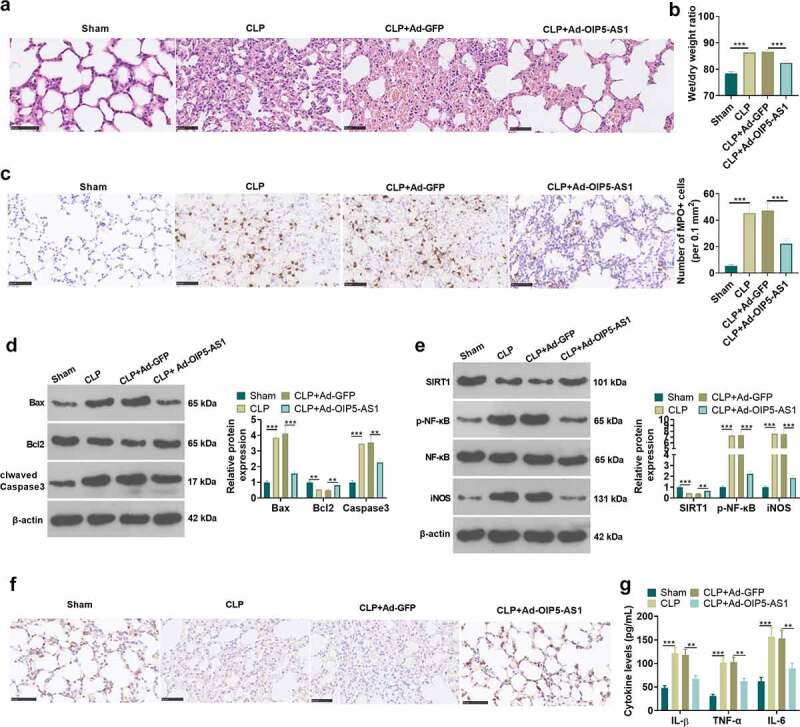
OIP5-AS1 overexpression weakened lung injury *in vivo*. Ad-GFP or Ad-OIP5-AS1 was injected into the SD rats via tail vein. CLP surgery was used to induce septic rat model. 48 hours after the construction of the mode, the lung tissues were isolated and subjected to further experiments. (a) The examination of the pathological changes in rat lung tissue of each group was conducted by HE staining method. (b) The dry/wet method was adopted for testing pulmonary edema. (c) ICH was used to evaluate MPO-labeled polymorphonuclear neutrophils (PMN). (d) The relative expression of apoptosis-related proteins Caspase3, Bax and Bcl2 related by apoptosis in each group was examined applying the Western blot method. (e) Western blot method was performed for the examination of the relative expression of SIRT1, p-NF-κB and iNOS. (f) SIRT1 expression in lung tissue was measured by immunohistochemical staining. (g) ELISA was applied for measuring the levels of related inflammatory factors IL-1β, TNFα and IL-6 in the lung homogenate supernatant. ** *P* < 0.01, *** *P* < 0.001. N = 5

To further probed the role of OIP5-AS1 on lung injury mediated by sepsis *in vivo*, we constructed a rat CLP model, and Ad-GFP or Ad-OIP5-AS1 was administered into the rats. HE staining was performed to test the pathological changes of lung tissue in each group of rats. The detection of the pulmonary edema was conducted using the dry/wet method. The data revealed that in CLP+ Ad-OIP5-AS1 group, the extent of the lung edema was alleviated, the quantity of bleeding of lung tissue was lessened and the cell nuclear staining showed an incomplete decline, in comparison with CLP+Ad-GFP group (*P* < 0.05, Figure 3a, b). Then ICH was used to evaluate MPO level in the lung tissues. The result showed that MPO-positive cells were significantly increased in the CLP group. With OIP5-AS1 overexpression, MPO-positively cells significantly reduced (*P* < 0.05, compared with CLP+Ad-GFP group, Figure 3c). Western blot method was respectively carried out for the examination of apoptosis-related proteins, including Caspase3, Bax, and Bcl2. It was demonstrated that overexpressing OIP5-AS1 lowered Bax and Caspase3 expressions, and led to an enhancement of Bcl2 expression (*P* < 0.05 compared with CLP+Ad-GFP group, Figure 3d). Meanwhile, OIP5-AS1 overexpression reversed the down-regulation of SIRT1 and the up-regulation of p-NF-κB and iNOS by CLP in rats (*P* < 0.05, Figure 3e). SIRT1 expression in lung tissue was further tested applying IHC. The results turned out that SIRT1 expression in CLP rats showed a decrease while OIP5-AS1 overexpression lifted SIRT1 expression (*P* < 0.05, Figure 3f). Additionally, we took ELISA to examine the level of inflammatory factors in each group in lung homogenate supernatant. It was discovered from the results that overexpressing OIP5-AS1 resulted in the down-regulation of the release of IL-1β, TNFα and IL-6 in rat lung tissue (*P* < 0.05, Figure 3g). The above results exhibit that OIP5-AS1 overexpression mitigates lung injury induced by CLP in rats.

### 3.4 MiR-128-3p overexpression facilitates lung epithelial cell injury and macrophage inflammation

**Figure 4.**
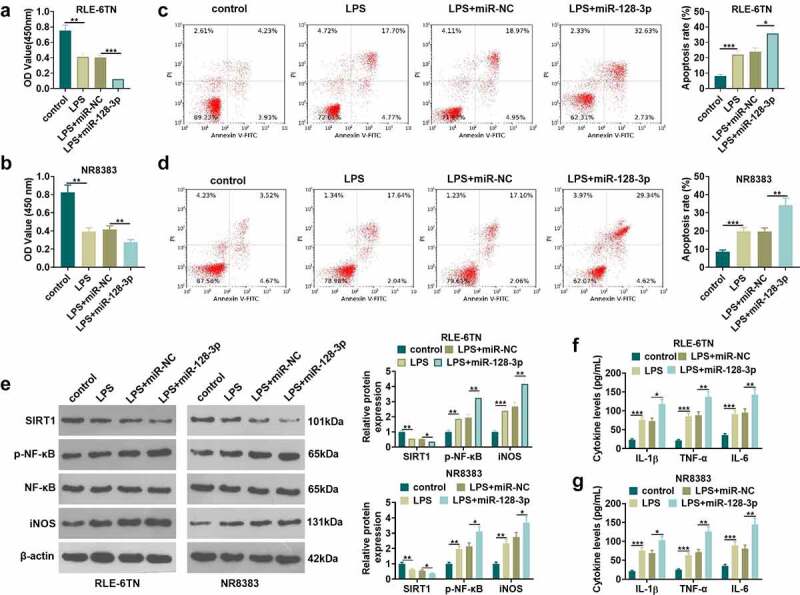
Overexpressing miR-128-3p accelerated lung macrophage inflammation and epithelial cell damage. RLE-6TN cells and NR8383 cells were transfected with miR-128-3p mimics or the negative control (miR-NC), and then both were treated with 100 ng/mL LPS for 6 hours. A-D. CCK8 assay and flow cytometry were conducted for the measurement of RLE-6TN cells and NR8383 cells cell viability (a-b) and apoptosis (c-d), respectively. (e) WB detected the expression of SIRT1, p-NF-κB and iNOS in RLE-6TN cells and NR8383 cells. F-G. We took ELISA to test the levels of IL-1β, TNFα and IL-6 in RLE-6TN cells and NR8383 cells. * *P* < 0.05, ** *P* < 0.01, *** *P* < 0.001. N = 3

Previous studies exhibited that miR-128-3p expression was elevated in sepsis rat model and sepsis cell model [31]. To further confirm miR-128-3p’s role in sepsis-induced ALI, the miR-128-5p mimics were transfected into RLE-6TN and NR8383 cells, following the treatment of LPS. CCK8 method and flow cytometry were respectively conducted for testing cell viability and apoptosis of RLE-6TN and NR8383 cells. The results showed that miR-128-3p overexpression lowered cell viability (*P* < 0.05 vs.LPS+miR-NC group, Figure 4a-b) and lifted apoptotic level (*P* < 0.05 vs.LPS+miR-NC group, Figure 4c-d). Next, western blot examined the expressions of SIRT1, p-NF-κB and iNOS in RLE-6TN and NR8383 cells. It was illustrated by the data that overexpressing miR-128-3p contributed to the reduction of SIRT1 expression and the elevation of p-NF-κB and iNOS expression (*P* < 0.05 vs.LPS+miR-NC group, Figure 4e). What is more, miR-128-3p overexpression in RLE-6TN and NR8383 cells remarkably heightened the levels of inflammatory factors (including IL-6, IL-1β and TNF-α) (*P* < 0.05 vs.LPS+miR-NC group, Figure 4f-g). These results display that the injury and inflammation of lung epithelial cell and macrophage are accelerated by miR-128-3p overexpression.

### 3.5 Overexpression of OIP5-AS1 attenuates the proinflammatory role-mediated by miR-128-3p

**Figure 5.**
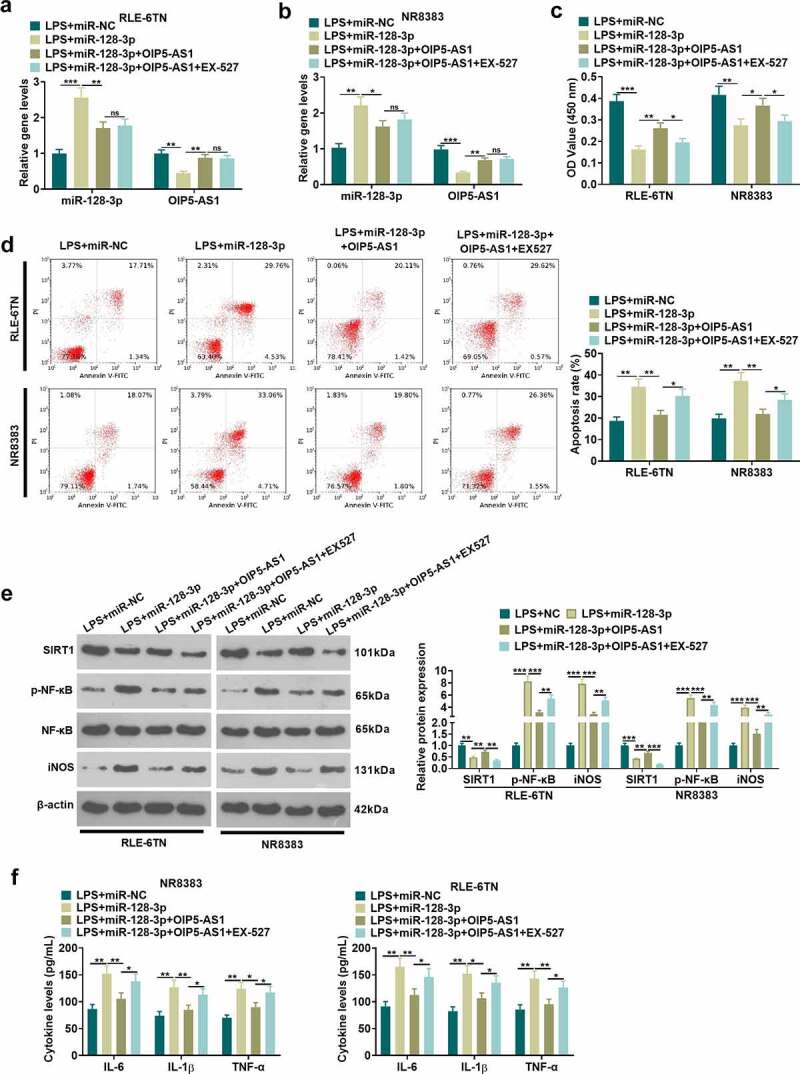
OIP5-AS1 overexpression attenuated miR-128-3p-mediated effects. RLE-6TN cells and NR8383 cells were transfected with miR-128-5p mimics, OIP5-AS1 overexpression plasmids, or treated with SIRT1 inhibitor (EX527, 100 nM), LPS (100 ng/mL) for 6 hours. A-B. We applied the qRT-PCR method for determining the relative expression of OIP5-AS1 and miR-128-3p in RLE-6TN and NR8383 cells. C-D. CCK8 method and flow cytometry were implemented for detecting cell viability and apoptosis of RLE-6TN and NR8383 cells. E. The relative expression of SIRT1, p-NF-κB and iNOS in RLE-6TN and NR8383 cells was examined employing Western blot. F. ELISA was adopted for the detection of the levels of IL-1β, TNFα and IL-6 in RLE-6TN and NR8383 cells. * *P* < 0.05, ** *P* < 0.01, *** *P* < 0.001. N = 3

To explore the OIP5-AS1-miR-128-3p-SIRT1 axis in sepsis-induced ALI, RLE-6TN and NR8383 were transfected with OIP5-AS1 overexpression plasmids, miR-128-5p mimics, and/or treated with SIRT1 inhibitor EX527. OIP5-AS1 and miR-128-3p relative expression in each group of RLE-6TN and NR8383 cells was examined using the qRT-PCR method. As the data showed, OIP5-AS1 overexpression attenuated miR-128-5p level, and promoted OIP5-AS1 expression (P<0.05 vs. LPS+miR-128-3p group). However, EX527 had no significantly effect on miR-128-3p and OIP5-AS1 expression (*P*>0.05, Figure 5a-b). We took CCK8 method to determine cell viability and utilized flow cytometry for the examination of apoptosis。As indicated by the results, OIP5-AS1 overexpression improved cell viability decreased and apoptosis increase (P<0.05 vs. LPS+miR-128-3p group). However, the SIRT1 inhibitor EX527 reduced cell viability and enhanced cell apoptosis compared with LPS+miR-128-3p+OIP5-AS1 group (*P*<0.05, Figure 5c, d)。The relative expression of SIRT1, p-NF-κB and iNOS in NR8383 cells was measured adopting the Western blot. It was exhibited that after overexpressing OIP5-AS1, SIRT1 expression showed an elevation while the expression of p-NF-κB and iNOS showed a decline. With the addition of EX527, SIRT1 expression was lessened, and the expression of p-NF-κB and iNOS was lifted (*P*<0.05, Figure 5e). ELISA data suggested that OIP5-AS1 overexpression mitigated the inflammatory cytokines (IL-6, IL-1β, TNF-α) release induced by miR-128-3p. The SIRT1 inhibitor EX527 enhanced IL-6, IL-1β, TNF-α levels in the supernatant (*P*<0.05 vs. LPS+miR-128-3p+OIP5-AS1 group, Figure 5f). It is manifested by the above results that overexpressing OIP5-AS1 attenuates LPS-induced inflammatory and apoptosis by miR-128-3p/SIRT1 axis.

### 3.6 MiR-128-3p targeted OIP5-AS1 and SIRT1

**Figure 6.**
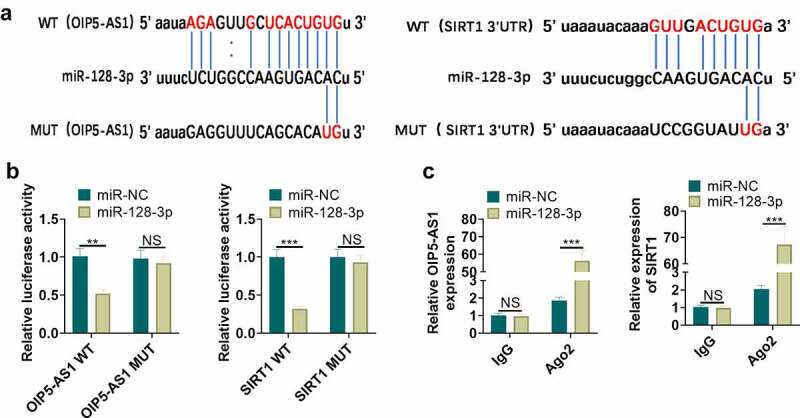
MiR-128-3p targeted OIP5-AS1 and SIRT1. A. The underlying binding sites between OIP5-AS1 and miR-128-3p, as well as miR-128-3p and SIRT1 predicted by bioinformatics were as shown. B. The dual-luciferase gene reporter method verified the targeting interrelationship between OIP5-AS1 and miR-128-3p, and miR-128-3p and SIRT1. C. The RIP method was utilized for validation of the targeting interrelationship between OIP5-AS1 and miR-128-3p, as well as miR-128-3p and SIRT1. ns*P*> 0.05, ** *P* < 0.01, *** *P* < 0.001. N = 3

For the purpose of probing the upstream and downstream molecular mechanisms of miR-128-3p, the analysis of miR-128-3p candidate targets was carried out through the StarBase database (http://starbase.sysu.edu.cn). We discovered the potential binding relationships between miR-128-3p and OIP5-AS1, as well as miR-128-3p and SIRT1 (Figure 6a). Identically, we validated the targeted binding interrelationship between miR-128-3p and OIP5-AS1, miR-128-3p and SIRT1 by the dual-luciferase gene reportor assay in NR8383 cells. The miR-128-3p mimic contributed to the reduction of the luciferase activity of cells transfected with OIP5-AS1-WT and SIRT1-WT vectors (*P < *0.05, Figure 6b), but had no considerable influence on that of cells transfected with OIP5-AS1-MUT and SIRT1-MUT vectors (*P > *0.05, Figure 6b). We took the RIP assay for further verification of the targeting relation between miR-128-3p and OIP5-AS1, and miR-128-3p and SIRT1. The OIP5-AS1 and SIRT1 enrichment were significantly increased after miR-128-5p mimics transfection (P < 0.05 compared with miR-NC group), suggesting that OIP5-AS1 targeted miR-128-3p and miR-128-3p targeted SIRT1 (Figure 6c). Taken together, miR-128-3p was targeted by OIP5-AS1, and targeted SIRT1.

## 4 Discussion

Sepsis is featured by the dysregulation of the host’s response to infection brings into the existence of life-threatening organ function damage [32]. During the occurrence and development of lung injury-mediated by sepsis, alveolar macrophages, which play a critical role in the control of bacteria, become activated and lead to immune system dysfunction in the body by secreting inflammatory cytokines, chemokines, damage mediators and pro-inflammatory cytokines, inducing apoptosis including bronchial epithelial cells, lung epithelial cells, lymphocytes [33–35]. As a consequence, the suppression of macrophage activation may serve as an underlying therapy for the treatment of lung injury mediated by sepsis.

Currently, it has been unveiled by many studies that various miRNAs exert a crucial influence on regulating macrophages. For example, miR-451 targets SIRT2 to regulate macrophage activation. MiR-451 overexpression represses the activation of macrophages and leads to a remarkable reduction of lung inflammation induced by triple-allergen [dust mite, ragweed, Aspergillus fumigatus (DRA)] [36]. In polymyositis and dermatomyositis, miR-146a, which targets regenerating family member 3 alpha (REG3A), restrains macrophage migration by lessening REG3A expression, sequentially weakening polymyositis and dermatomyositis [37]. LncRNA, as a competitive endogenous RNA (ceRNA), regulates sepsis by the competitive regulation of specific miRNA expression. For instance, in acute kidney injury in septic rats, the competitive binding of lncRNA HOX transcript antisense RNA (HOTAIR) and miR-34a results in the regulation of Bcl-2 expression, and overexpressing HOTAIR restrains apoptosis of renal tissues, thereby lowering acute kidney injury in septic rats [38]. In this paper, OIP5-AS1 shows a shortened expression while miR-128-3p is highly expressed in sepsis rat model and cell model. OIP5-AS1 targets miR-128-3p to repress the inflammatory response of both *in vitro* and *in vivo*, suggesting that OIP5-AS1-miR-128-5p axis is a potential therapeutic target against sepsis.

As a conserved family of NAD+ dependent histone deacetylases and mono-ADP-ribosyltransferases, SIRTs have been found with potent anti-inflammatory effects by targeting histones, transcription factors and coregulators to adapt gene expression to the cellular energy stat [39]. SIRT1 is a part of the histone deacetylases class, and scholars have revealed that SIRT1 suppresses the release of inflammatory factors and lessens tissue damage during inflammation [40,41]. During sepsis, NF-κB phosphorylation levels increase and translocate into the nucleus, promoting the transcription of inflammatory factors [42]. SIRT1 can inhibit I-κB phosphorylation, thus inactivating NF-κB pathway and exerting anti-inflammatory effect [43]. As a downstream molecule of NF- κB pathway, inducible nitric oxide synthase (iNOS) has a certain extent of influence on inflammatory reaction and oxidative stress [44]. A large amount of NO is produced by catalysis of iNOS induced by diversified cytokines including interleukin and lipopolysaccharide, consequently accelerating cell apoptosis [45]. SIRT1/NF-κB/iNOS axis takes part in regulating multiple inflammatory diseases. Taking pseudodoginsengenin DQ as an example, it leads to the reduction of TNF-1 and iNOS expression through Sirt1/NF-κB and Caspase signal transduction pathways, resulting in the protection against nephrotoxicity induced by cisplatin [46]. Asiatic acid weakens iNOS expression induced by LPS and the expression of IL-6, IL-1β, TNFα and other pro-inflammatory factors by regulating Sirt1/NF-κB signaling pathway, alleviating the microglial neuroinflammation [47]. In the present study, we have unveiled that in rats and NR8383 and RLE-6TN cells mediated by sepsis, SIRT1 relative expression shows a decrease, and the relative expression of p-NF-κB and iNOS is increased. Serving as an endogenous RNA, OIP5-AS1 plays an inhibited role in miR-128-3p, which targets SIRT1. OIP5-AS1 overexpression facilitates SIRT1 expression, restrains the phosphorylation of NF-κB in NR8383 and RLE-6TN cells, and down-regulates iNOS expression. Thus, OIP5-AS1 shows potent inhibition of the of pro-inflammatory factors release both *in vivo* and *in vitro.*

Generally speaking, our study has manifested that in sepsis-mediated lung injury, OIP5-AS1/miR-128-3p/SIRT1 plays a vital role by suppression of phosphorylation of NF-κB and the release of pro-inflammatory factors. This study suggests that OIP5-AS1 may serve as an underlying targeting molecule for the treatment of lung injury mediated by sepsis. However, further experiments were also need to confirm the regulatory axis of OIP5-AS1/miR-128-3p/SIRT1 in sepsis.

## Data Availability

The data sets used and analyzed during the current study are available from the corresponding author on reasonable request.

## Abbreviations

cDNA: complementary DNA; ceRNA: competitive endogenous RNA; CLP: cecal ligation and puncture; CRNDE: colorectal neoplasia differentially expressed; DRA: dust mite, ragweed, Aspergillus fumigatus; EGF: epidermal growth factor; eIF2a: eukaryotictranslation initiation factor 2A; H2O2: hydrogen peroxide; HOTAIR: HOX transcript antisense RNA; HRP: horseradish peroxidase; IGF: insulin-like growth factor; IHC: Immunohistochemistry; IL-1β: interleukin-1β; IL-6: interleukin-6; IL-8: interleukin-8; iNOS: inducible nitric oxide synthase; KCNQ1OT1: KCNQ1 opposite strand/antisense transcript1; lncRNA OIP5-AS1:long non-coding RNA OIP5 antisense RNA 1; LPS: lipopolysaccharide; MIAT: myocardial infarction associated transcript; miRNAs: MicroRNAs; MUT: mutant; NC: negative control;NF-κB: nuclear factor kappa B; PBS: phosphate buffered saline; PVDF: polyvinylidene fluoride; qRT-PCR: quantitative reverse transcription-polymerase chain reaction; REG3A: regenerating family member 3alpha; RIP: RNA immunoprecipitation; SD: Sprague-Dawley; SIRT1: sirtuin1; TNF-α: tumor necrosis factor-α; WT: wild type; XIAP: X-linked inhibitor of apoptosis.
